# Draft genome sequences of eight biofilm-forming *Vibrio* spp. strains isolated from marine ecosystems

**DOI:** 10.1128/mra.00199-26

**Published:** 2026-08-24

**Authors:** Cécile Berdous, Dorothee Gotz, Marie-Françoise Noirot-Gros, Eric Leclercq, Virgile Guéneau, Romain Briandet

**Affiliations:** 1 Université Paris-Saclay, INRAE, AgroParisTech, Micalis Institute, Jouy-en-Josas, France; 2 LabCom Biofilm1Health, Lallemand SAS & Micalis Institute, INRAE, AgroParisTech, Université Paris-Saclay 27048, Jouy-en-Josas, France; 3 Lallemand SAS 483681 , Blagnac, France; University of Wisconsin-Madison, Madison, Wisconsin, USA

**Keywords:** *Vibrio*, virulence factors, biofilms, marine microbiology

## Abstract

We report the genome sequences of eight environmental biofilm-forming *Vibrio* strains isolated from diverse marine environments. Genome analysis revealed the presence of multiple antimicrobial resistance and virulence-associated genes, providing a valuable resource for future comparative and ecological studies of environmental *Vibrio* species.

## ANNOUNCEMENT

Environmental *Vibrio* species are ubiquitous in marine ecosystems, colonizing biotic and abiotic surfaces through biofilm formation (1). Several species, including *Vibrio parahaemolyticus* (2), *Vibrio alginolyticus* (3), and *Vibrio antiquarius* (4), are ecologically versatile and act as opportunistic pathogens in marine animals and, in some cases, humans. The combination of biofilm-associated lifestyles, genetic plasticity, and strain-specific virulence and resistance traits raises One Health concerns.

Here, we report the draft genome sequences of eight environmental *Vibrio* strains isolated between 2007 and 2012 from geographically distinct marine habitats, spanning Mayotte (Indian Ocean), the Atlantic coast (Asturias, Spain), and the Black Sea (Romania), from seawater, sediments, and marine invertebrates.

Samples were vortexed, coarsely filtered to remove large particles before plating onto Marine Agar Difco, and incubated at 30°C for 24 h. Colonies were stored in glycerol at −80°C. The ones identified as *Vibrio* spp. by 16S rRNA gene sequencing were selected for whole-genome sequencing (WGS). Overnight cultures grown in marine broth (30°C, ~18 h) were used for DNA extraction using the New England Biolabs Monarch Genomic DNA Purification Kit (New England Biolabs, #T3010S) following the manufacturer’s instructions. DNA concentrations were measured using the Qubit dsDNA quantification assay kits.

WGS was performed by Eurofins Genomics Europe on an Illumina NovaSeq X Plus System platform with paired-end (2 × 150 bp) reads, generating approximately 5 million paired-end reads per strain, corresponding to an average sequencing depth exceeding 100×. Raw reads were processed on the Inrae Migale Galaxy platform using FastQC (v.0.12.1) (5) for quality control, FastP (v.0.23.2) (6) for trimming, Unicycler (v.0.5.1) (7) for *de novo* assembly, QUAST (v.5.0.2) (8) for assembly quality assessment, and Prokka (v.1.14.6) (9) for annotation. Species identification relied on PubMLST’s rMLST database (10). Antimicrobial resistance genes were detected using StarAMR (v.0.11.0) (11), which screens contigs against ResFinder, PlasmidFinder, and PointFinder databases (12–14), while toxin genes were identified by comparison with the VFDB database (15). Assembly statistics are summarized in Table 1. Genome sizes ranged from 5.0 to 5.5 Mb, with guanine + cytosine (G + C) contents between 44.5 and 45.4%, consistent with environmental *Vibrio* species.

**TABLE 1:** Draft genome assembly statistics of eight *Vibrio* spp. isolates, ordered by their strain codes^*a*^

Strains	AQP6078	AQP8982	AQP8990	AQP10324	AQP8714	AQP8996	AQP10327	AQP10405
Accession numbers	SRR37455625	SRR37455624	SRR37455623	SRR37455622	SRR37455621	SRR37455620	SRR37455619	SRR37455618
Species	*V. parahaemolyticus*	*V. parahaemolyticus*	*V. parahaemolyticus*	*V. parahaemolyticus*	*V. alginolyticus*	*V. alginolyticus*	*V. alginolyticus*	*V. antiquarius*
Origin and year of isolation	Marine cave, Asturias, Spain Sea anemone, *Bunodactis* sp. (Actiniaria), 2007	Pinacle Mtsamoudou, Nord-Ouest, Mayotte *Sertella* sp. bryozoan from coral reef, 2011	Pinacle Mtsamoudou, Nord-Ouest, Mayotte *Sertella* sp. bryozoan from coral reef, 2011	Black Sea, Navodari, Romania Sediment, from environment of concrete blocks in sandy sea floor, 2012	Parc Saziley site Pointe Crocodile, Mayotte sediment from mangrove area, 2011	Parc Saziley face Nord, Mayotte Ferrobacteria colony over wreck, 2011	Black Sea, Navodari, Romania sediment, from environment of blocks in sandy sea floor, 2012	Black Sea, Romania sediment, from environment of blocks in Agigea channel, 2012
Quality-filtered reads and their representative percentage	4,573,258 (99.9%)	15,595,462 (99.5%)	10,989,090 (99.7%)	10,186,053 (99.9%)	3,662,819 (99.7%)	3,924,616 (99.9%)	12,101,819 (99.9%)	10,681,125 (99.91%)
Completeness	100.00	100.00	100.00	100.00	99.86	100.00	99.86	100.00
Contamination	0.01	1.25	0.00	0.14	0.05	0.11	0.05	0.08
Genome size (bp)	5,049,751	5,355,321	5,509,714	5,130,870	5,118,766	5,019,067	5,117,601	5,153,574
No. of contigs	81	100	83	54	69	55	73	55
GC content (%)	45.38%	45.13%	45.17%	45.19%	44.54%	44.57%	44.54%	44.68%
N50 (bp)	603,104	519,081	341,712	481,025	298,975	566,017	298,975	808,777
tRNA	96	80	92	82	74	80	74	81
rRNA	3	7	3	3	4	3	4	2
Protein-coding sequences (CDS)	4566	4460	5057	4626	4569	4460	4643	4673
Antibiotic resistance genes	*tet(34)*, *blaCARB-18*, *tet(35*)	*fos, blaCARB*, *tet(35)*, *tet(34)*, *tet(K*)	*qnrVC10*, *tet(34)*, *tet(35)*, *blaCARB-18*	*tet(35)*, *tet(34)*, *blaCARB-18*	*tet(34)*, *blaCARB-42*, *tet(35*)	*tet(34)*, *tet(35)*, *blaCARB*	*tet(34)*, *blaCARB-42*, *tet(35*)	*blaCARB*, *fos*, *tet(34)*, *tet(35)*, *qnrS*
Toxin genes	*tdh*, *toxS*	*tdh*	*tdh1*, *tdh2*, *toxS*	*tdh*, *toxS*	*tdh1*, *toxS*	*tdh1*, *tdh2*, *tdh3*	*tdh1*, *toxS*	*tdh1*, *tdh2*
Biofilms biovolume (µm³/µm²)	13.9 ± 1.3	17.5 ± 3.9	12.7 ± 4.2	15.7 ± 2.3	12.0 ± 1.8	10.1 ± 3.2	8.2 ± 1.8	7.4 ± 0.8

^*a*^ The analysis includes genome features, annotated antibiotic resistance genes, toxin genes, and quantitative biofilm phenotypes expressed as surface-normalized biovolume (means ± SD in µm³/µm²).

All strains harbored multiple antimicrobial resistance genes, including those associated with β-lactams and tetracyclines (16). PlasmidFinder analysis identified a single replicon, rep7a, exclusively in strain AQPB8982 associated with tetracycline resistance (accession AB037671). The *vgrG1* encoding a T6SS structural component involved in interbacterial competition (17) was detected in all eight strains. Genes *toxS* and *tdh* were also identified in selected strains (Table 1), encoding the ToxR/ToxS regulatory system and the thermostable direct hemolysin, both key virulence determinants in *Vibrio* spp. (18, 19). The biofilm-forming capacity was quantified by confocal laser scanning microscopy and BiofilmQ image analysis (20, 21) after 24 h growth in marine broth at 30°C. All strains formed biofilms, with biovolume values exceeding 7 µm³/µm² (Table 1).

These genome sequences provide a valuable resource for future comparative genomic analyses aimed at elucidating the genetic determinants of biofilm formation, environmental persistence, and the emergence of pathogenic traits in marine *Vibrio* populations.

## Data Availability

This Whole Genome Shotgun project has been deposited at DDBJ/ENA/GenBank under accession numbers JBVVWO000000000, JBVVWN000000000, JBVVWM000000000, JBVVWL000000000, JBVVWK000000000, JBVVWJ000000000, JBVVWI000000000, and JBVVWH000000000 for strains AQP6078, AQP8982, AQP8990, AQP10324, AQP8714, AQP8996, AQP10327, and AQP10405, respectively. The draft genome assembly described on this paper can be found on NCBI under BioProject number PRJNA1428756 and SRA numbers SRR37455625, SRR37455624, SRR37455623, SRR37455622, SRR37455621, SRR37455620, SRR37455619, and SRR37455618 for strains AQP6078, AQP8982, AQP8990, AQP10324, AQP8714, AQP8996, AQP10327, and AQP10405, respectively.
